# Cellular Adaptation Facilitates Sparse and Reliable Coding in Sensory Pathways

**DOI:** 10.1371/journal.pcbi.1003251

**Published:** 2013-10-03

**Authors:** Farzad Farkhooi, Anja Froese, Eilif Muller, Randolf Menzel, Martin P. Nawrot

**Affiliations:** 1 Neuroinformatics & Theoretical Neuroscience, Freie Universität Berlin, and Bernstein Center for Computational Neuroscience Berlin, Berlin, Germany; 2 Institute für Biologie-Neurobiologie, Freie Universität Berlin, Berlin, Germany; 3 Blue Brain Project, École Polytechnique Fédérale de Lausanne, Lausanne, Switzerland; École Normale Supérieure, College de France, CNRS, France

## Abstract

Most neurons in peripheral sensory pathways initially respond vigorously when a preferred stimulus is presented, but adapt as stimulation continues. It is unclear how this phenomenon affects stimulus coding in the later stages of sensory processing. Here, we show that a temporally sparse and reliable stimulus representation develops naturally in sequential stages of a sensory network with adapting neurons. As a modeling framework we employ a mean-field approach together with an adaptive population density treatment, accompanied by numerical simulations of spiking neural networks. We find that cellular adaptation plays a critical role in the dynamic reduction of the trial-by-trial variability of cortical spike responses by transiently suppressing self-generated fast fluctuations in the cortical balanced network. This provides an explanation for a widespread cortical phenomenon by a simple mechanism. We further show that in the insect olfactory system cellular adaptation is sufficient to explain the emergence of the temporally sparse and reliable stimulus representation in the mushroom body. Our results reveal a generic, biophysically plausible mechanism that can explain the emergence of a temporally sparse and reliable stimulus representation within a sequential processing architecture.

## Author Summary

Many lines of evidence suggest that few spikes carry the relevant stimulus information at later stages of sensory processing. Yet mechanisms for the emergence of a robust and temporally sparse sensory representation remain elusive. Here, we introduce an idea in which a temporal sparse and reliable stimulus representation develops naturally in spiking networks. It combines principles of signal propagation with the commonly observed mechanism of neuronal firing rate adaptation. Using a stringent numerical and mathematical approach, we show how a dense rate code at the periphery translates into a temporal sparse representation in the cortical network. At the same time, it dynamically suppresses trial-by-trial variability, matching experimental observations in sensory cortices. Computational modelling of the insects olfactory pathway suggests that the same principle underlies the prominent example of temporal sparse coding in the mushroom body. Our results reveal a computational principle that relates neuronal firing rate adaptation to temporal sparse coding and variability suppression in nervous systems.

## Introduction

The phenomenon of spike-frequency adaptation (SFA) [1], which is also known as spike-rate adaptation, is a fundamental process in nervous systems that attenuates neuronal stimulus responses to a lower level following an initial high firing. This process can be mediated by different cell-intrinsic mechanisms that involve a spike-triggered self-inhibition, and which can operate in a wide range of time scales [2]–[4]. These mechanisms are probably related to the early evolution of the excitable membrane [5]–[7] and are common to vertebrate and invertebrate neurons, both in the peripheral and central nervous system [8]. Nonetheless, the functional consequences of SFA in peripheral stages of sensory processing on the stimuli representation in later network stages remain unclear. For instance, light adaptation in photoreceptors strongly shapes their responses [9], [10] and affects stimulus information in second-order neurons [11]. In a seminal work by Hecht and colleagues [12], it was shown that during dark adaptation, 10 or less photon absorptions in the retina were sufficient to give a sensation of light within a millisecond of exposure and the response variability could be largely accounted for by quantum fluctuations. This is an interesting empirical result, and still it is theoretically puzzling that the intrinsic noise of the nervous system [13] has only little influence on the detection of such an extremely weak stimulus. A proposal by Barlow [14] suggested that successive processing in sensory neural pathways decrements the number of response spikes and therefore the informativeness of each spike increases while the level of noise decreases. However, it remains unclear how such temporally sparse spike responses can reliably encode information in the face of the immense cortical variability [15] and the sensitivity of cortical networks to small perturbations [16], [17].

The widespread phenomenon of a dynamically suppressed trial-by-trial response variability in sensory and motor cortices [18]–[20] along with a sparse representation [21], [22] hints at an increased reliability of the underlying neuronal code and may facilitate the perception of weak stimuli. However, the prevailing cortical network models of randomly connected spiking neurons, where the balance of excitation and inhibition is quickly reinstated within milliseconds after the arrival of an excitatory afferent input, do not capture this dynamic [16], [17], [23]–[25]. Recent numerical observations suggest that a clustered topology of the balanced network [26] or attractor networks with multi-stability [27] provide possible explanations for suppressing cortical variability during afferent stimulation.

In this study, we introduce an alternative and unified description in which a temporally sparse stimulus representation and the transient increase of response reliability emerge naturally. Our approach exploits the functional consequences of SFA in multi-stage network processing. Here, we show that the SFA mechanism introduces a dynamical non-linearity in the transfer function of neurons. Subsequently, the response onset becomes progressively sparser when transmitted across successive processing stages. We use a rigorous master equation description of neuronal ensembles [28]–[30] and numerical network simulations to arrive at the main result that the self-regulating effect of SFA causes a stimulus-triggered reduction of firing variability by modulating the average inhibition in the balanced cortical network. In this manner the temporally sparse representation is accompanied by an increased response reliability. We further utilize this theoretical framework to demonstrate the generality of this effect in a highly structured network model of insect olfactory sensory processing, where sequential neuronal adaptation readily explains the ubiquitously observed sparse and precisely timed stimulus response spikes at the level of the so-called Kenyon cells [31]–[34]. Our experimental results qualitatively supports this theoretical prediction.

## Results

### Temporal sparseness emerges in successive adapting populations

**Figure 1:**
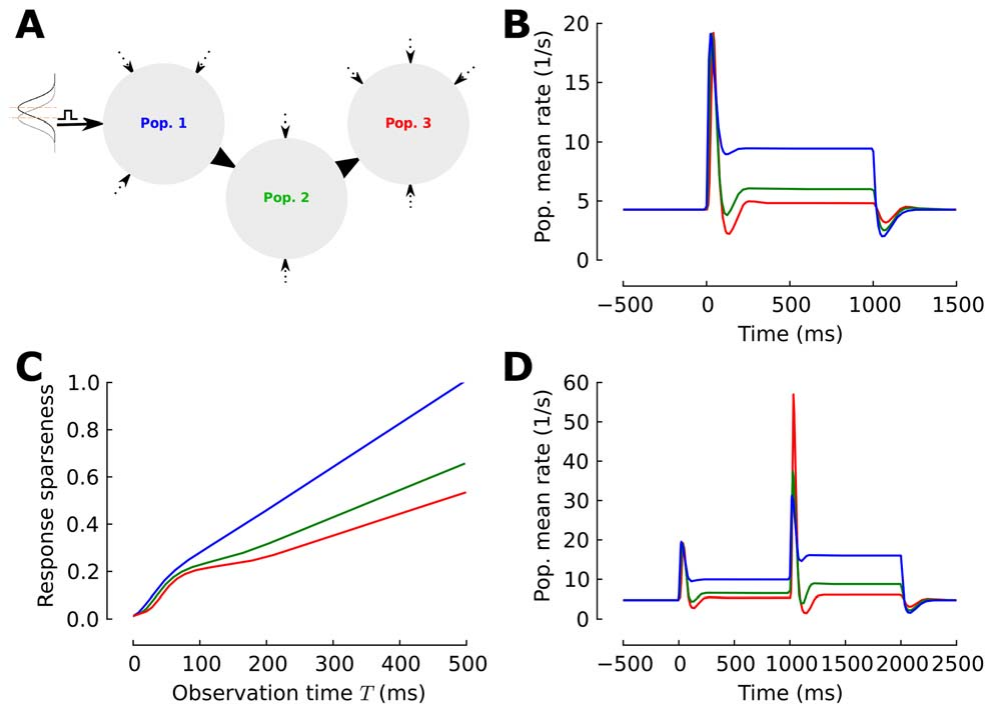
Neuronal adaptation in the multi-stage processing network. (**A**) Schematic illustration of a three-layered model of an adaptive pathway of sensory processing. The network consists of three consecutive adaptive populations. Each population receives sensory input from an afferent source (black arrows) and independent constant background excitation (dashed arrow). Input is modeled by a Gaussian density and a sensory stimulus presented to the first population is modeled by an increase in the mean input value. (**B**) Response profiles. The evoked state consists of a phasic-tonic response in all populations. The tonic response level is decremented across the consecutive populations. (**C**) Temporal sparseness 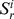 is measured by the integral over the firing rate and normalized by the average spike count at 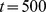
ms in the first population. Responses become progressively sparser as the stimulus propagates into the network. (**D**) Secondary response profiles. The additional jump increase in stimulus strength at 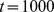
ms during the evoked state of the first stimulus results in a secondary phasic response in all populations with an amplitude overshoot in the 2nd and 3rd population.

To examine how successive adapting populations can achieve temporal sparseness, first we mathematically analyzed a sequence of neuronal ensembles (**Figure 1A**), where each ensemble exhibits a generic model of mean firing rate adaptation by means of a slow negative self-feedback [2], [28], [35] (**Materials and Methods**). This sequence of neuronal ensembles should be viewed as a caricature for distinct stages in the pathway of sensory processing. For instance, in the mammalian olfactory system the sensory pathway involves several stages from the olfactory sensory neurons to the olfactory bulb, the piriform cortex, and then to higher cortical areas (**Figure 1A**).

The mean firing rate in the steady-state of a single adaptive population can be obtained by solving the rate consistency equation, 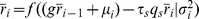, where 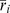 is the equilibrium mean firing rate of the 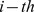 population, 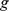, 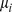 and 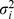 are coupling strength, mean and variance of the total input into the population, respectively, 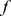 is the response function (input-output transfer function, or 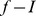 curve) of the population mean activity, 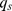 is the quantal conductance of the adaptation mechanism per unit of firing rate, and 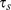 is the adaptation relaxation time constant [2], [28], [35]. The firing rate model assumes that individual neurons spike with Poisson statistics, and that the adaptation level only affects mean input into neurons, resulting in a change to the steady-state mean firing rate. It is known that any sufficiently slow modulation (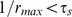) linearizes the steady-state solution, 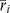, due to the self-inhibitory feedback being proportional to the firing rate (**see Materials and Methods**) [36].

Here, for simplicity, we studied the case where all populations in the network exhibit the same initial steady-state rate. This is achieved by adjustment of a constant background input to population 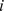, given 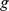 (doted arrows, **Figure 1A**), resembling the stimulus irrelevant interactions in the network. All populations are coupled by the same strength 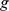. First, we calculated the average firing rate dynamics of the populations' responses following a step increase in the mean input to the first layer (black arrow, **Figure 1A**). By solving the dynamics of the mean firing rate and adaptation level concurrently, we obtained the mean-field approximation of the populations' firing rates (**Materials and Methods**). As it is typical for adapting neurons, the responses of each population consisted of a fast transient following stimulus onset before it converges to the new steady-state (tonic response part) with a stable *focus* (**Materials and Methods**). The **Figure 1B** shows the mean firing rate of three consecutive populations. The phasic response to the step increase in the input is preserved across stages. However, the tonic response becomes increasingly suppressed in the later stages (**Figure 1B**). This phenomenon is a general feature of successive adaptive neuronal populations with a non-linear transfer function 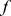 which, is linearized in steady-state due to adaptation negative feedback [36]. This result emerges as the change in the 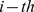 population mean rate that can be determined by solving the rate consistency equation now for a step change in the input 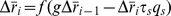. The necessary condition for the suppression of the steady-state responses is a sufficiently strong adaptation (**Materials and Methods**). It is worth to note that the populations exhibit under-shoots after the offset of the stimulus (**Figure 1B**). This is due to the adaptation level that accumulated during the evoked state.

The result in this sub-section (**Figure 1B–C**) was established with a current based leaky integrated-and-fire response function. However, the analysis presented here extends to the majority of neuronal transfer functions since the stability and linearity of the adapted steady-states are granted for many biophysical transfer functions [35], [36]. This simple effect leads to a progressively sparser representation across successive stage of a generic feed-forward adaptive processing. We assess temporal sparseness by computing the time-dependent integral 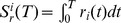, where 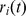 is the mean firing rate of population 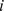 and 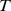 is the increasing observation time window. Normalization of this measure by the spike count in the first population 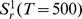 indicates that responses in the later stages of the adaptive network are temporally sparser (**Figure 1C**). This is expressed in the sharp increase of the rate integral during the transient response, whereas the first population integral shows an almost constant increase in the number of spikes.

Does the suppression of the adapted response level impair the information about the presence of the stimulus? To explore this, we studied a secondary increase in stimulus strengths with an equal magnitude, after 1 second, when the network has relaxed to the evoked equilibrium (**Figure 1D**). The secondary stimulus jump induced a secondary phasic response of comparable magnitude in the first population (**Figure 1D**). However, in the later populations this jump evoked an increased peak rate in the phasic response (**Figure 1D**). Notably, the coupling factor 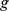 between the populations shapes this phenomena. Here, we adjusted 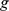 to achieve an equal onset response magnitude across the populations for the first stimulus jump at 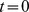, and a slight increase in the population onset response in the first population is amplified in the later stages. This is due to the fact that the later stages accumulated less adaptation in their evoked steady-state (the level of adaptation is proportional to mean firing rate). Importantly, this result confirms that the sustained presence of the stimulus is indeed stored at the level of cellular adaptation [37], even though it is not reflected in the firing rate of the last population (**Materials and Methods**). Therefore, regardless of the absolute amplitude of responses, the relative relation between secondary and initial onset keeps increasing across layers. This type of secondary overshoot is also experimentally known as *sensory sensitization* or *response amplification*, where an additional increase in the stimulus strength significantly enhances the responsiveness of later stages after the network converged to an adapted steady-state [10], [11], [38], [39].

### Adaptation increases response reliability in the cortical network

The mean firing rate approach as above is insufficient to determine how reliable the observed response transients are across repeated simulations. In a spiking model of neo-cortex (the balanced network), self-generating recurrent fluctuations strongly dominate the dynamics of interactions and produce highly irregular and variable activity [24]. This prevailing cortical model suggests that balance of excitation and inhibition is quickly reinstated within milliseconds after the onset of an excitatory input and adjusts the network fluctuations level [23]. Therefore, it has been questioned whether a few temporarily meaningful action potentials could reliably encode the presence of a stimulus [16].

**Figure 2:**
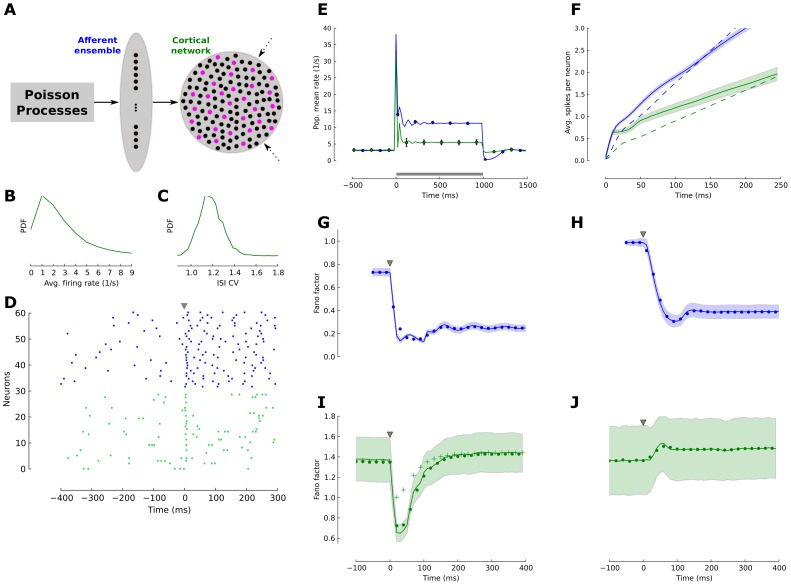
Reliability of a temporally sparse code in the balanced cortical network. (**A**) Schematic of a two-layer model of sub-cortical and early cortical sensory processing. The afferent ensemble (blue) consists of 4,000 independent neurons, and each neuron projects to 1% of the neurons in the cortical network (green). The cortical network is a balanced network in the asynchronous and irregular state with random connectivity. In both populations black circles represent excitatory neurons and magenta circles represent inhibitory cells. (**B**) The distribution of firing rates across neurons in the cortical network is fat-tailed and the average firing rate is approximately 3 Hz. (**C**) The distribution of the coefficient of variation (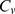) across neurons in the balanced cortical network confirms irregular spiking. (**D**) Spike raster plot for a sample set of 30 afferent neurons (blue dots) and 30 excitatory cortical neurons (green dots). At 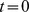 (gray triangle) the stimulus presentation starts. (**E**) Population averaged firing for both network stages. The simulation (solid lines) follows the calculated ensemble average predicted by the adaptive density treatment (black circles). The firing rate in the simulated network is estimated with a 20 ms bin size. (**F**) Number of spikes per neuron 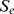 after stimulus onset (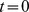) for the adaptive network (solid lines) and the weakly adaptive control network (dashed lines). Cortical excitatory neurons (green) produced less spikes than neurons in the earlier stage (afferent ensemble, blue). The shaded area indicates the standard deviation across neurons. (**G,H**) Fano factor dynamics of the afferent ensemble in the network with strongly adapting neurons (G, 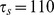
ms) and in the weakly-adaptive (H, 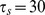
ms) network, estimated across 200 trials in a 50 ms window and a sliding of 10 ms for the ensemble network with adaptation. The black circles indicate the theoretical value of the Fano factor computed by adaptive density treatment and shaded area is the standard deviation of the Fano factor across neurons in the network. (**I**) The Fano factor of strongly adaptive neurons in cortical balanced network reduced transiently during the initial phasic response part. The crosses show the adaptive cortical ensemble Fano factor for the case where the afferent ensemble neurons were modeled as a Poisson process with the same steady-state firing rate and without adaption. (**J**) The Fano factor in the weakly adaptive cortical network did not exhibit a reduction during stimulation.

To investigate the reliability of adaptive mapping from a dense stimulus to a sparse cortical spike response across successive processing stages we employed the adaptive population density formalisms [28], [29] (**Materials and Methods**) along with numerical network simulations. We embedded a two-layered sensory network with an afferent ensemble projecting to a cortical network (**Figure 2A**). The afferent ensemble consisted of 4,000 adaptive neurons that included voltage dynamics, conductance-based synapses, and spike-induced adaptation [28]. It resembles the sub-cortical sensory processing and each neuron in the afferent ensemble projects randomly to 1% of the neurons in the cortical network. This is a large circuit of the balanced network (**Figure 2A**) with 10,000 excitatory and 2,500 inhibitory neurons with a typical random diluted connectivity of 1%. The spiking neuron model in the cortical network again includes voltage dynamics, conductance-based synapses, and spike-induced adaptation [28]. All neurons are alike and parameters are given in Table 3 in Muller et al. [28]. With appropriate adjustment of the synaptic weights, the cortical network operates in a globally balanced manner, producing irregular, asynchronous activity [24], [40], [41]. The distribution of firing rates for the network approximates a power-law density [42] with an average firing rate of 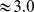
Hz (**Figure 2B**) and the coefficients of variation (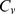) for the inter-spike intervals are centered at a value slightly greater than unity (**Figure 2C**) indicating the globally balanced and irregular state of the network [41]. Noteworthy is that the activity of neurons in both stages is fairly incoherent and spiking in each sub-network is independent. Therefore, one can apply an adiabatic elimination of the fast variables and formulate a population density description where a detailed neuron model reduces to a stochastic point process [28], [29] that provides an analytical approximation of the spiking dynamics and helps understanding the network simulation results in this section. This framework allows for a detailed study of a large and incoherent network without the need of numerical simulations (**Materials and Methods**).

The background input is modelled as a set of independent Poisson processes that drive both sub-networks (dashed arrows, **Figure 2A**). The stimulus dependent input is an increase in the intensity of the Poisson input into the afferent ensemble (solid arrow, **Figure 2A**). Before the stimulus became active at time 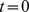, a typical neuron showed an irregular spiking activity in both network stages (**Figure 2B–D**). Whenever a sufficiently strong stimulus is applied all neurons in the afferent ensemble exhibited a transient response before the population mean firing rate converges back to a new level of steady-state (**Figure 2D,E**). The population firing rate of neurons in the cortical network also exhibited a transient evoked response (**Figure 2D,E**). However, in the balanced network individual neurons are heterogeneous in their responses (**Figure 2D**), since the number of inputs from afferent and recurrent connectivity are random. In contrast to the rate model in the previous section where individual neurons were assumed to spike in a Poissonian manner, the adaptive neuron model in the neural network simulation operates far away for this assumption since the adaptation endows a long lasting memory effect on the spike times [28], [29] that extends beyond the last spike. The time constant of this memory is determined by the time constant of adaptation (*τ_s_* = 110 ms). This non-renewal statistics determines the shape of the transient component of the population response in **Figure 2E**. The spiking irregularity shows that the evoked state in the afferent ensemble is more regular than its background. The balanced network still exhibits a fairly irregular spiking and its average 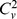 stays approximately constant slightly above 1 (**Figure 2D**). The population firing rate in the numerical simulations (solid line, **Figure 2E**) follow well the adaptive population density treatment (filled circles, **Figure 2E**).

To measure the effect of neuronal adaptation on the temporal sparseness, we again computed the number of spikes per neuron after the stimulus onset, 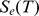. We compare our standard adaptive network with an adaptation time constant of *τ_s_* = 110 ms (solid lines, **Figure 2F**) to a weakly-adaptive control network (*τ_s_* = 30 ms; dashed lines, **Figure 2F**). Note, that the adaptation time constant in the weakly adaptive network is about equal to the membrane time constant and therefore plays a minor role for the network dynamics. It showed that both sub-networks generated sharp population level phasic response, which in the case of the cortical network evoked a single sharply timed spike within the first 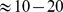
ms in a subset of neurons (**Figure 2B,F**). In the control case, the response is non-sparse and response spikes are distributed throughout the stimulus period (dashed lines, **Figure 2F**). Overall, strong adaptation reduces the total number of stimulus-induced action potentials per neuron and concentrates their occurrence within an initial brief phasic response part following the fast change in the stimulus. This temporal sparseness is reflected in the cumulative number of spikes per neuron (**Figure 2F**) which increases sharply. Thus, in accordance with the results of the rate-based model in the previous section, one can conclude that the sequence of adaptive processing accounts for the emergence of a temporally sparse stimulus representation in a cortical population.

We also estimated the fraction of neurons that significantly changed their number of spikes after stimulus onset. By construction, all cells in the afferent ensemble, both in adaptive and weakly-adaptive cases, produce a significant response. However, neurons in the cortical layer are far more selective. In the weakly-adaptive network 58% of all neurons responded significantly. In the adaptive network this number drops to 36%. This is calculated by comparing the count distribution across trials in 200 ms windows before and after the stimulus onset (Wilcoxon rank sum test, p-value = 0.01).

To reveal the effect of adaptation on the response variability, we employed the time-resolved Fano factor [20], 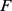, which measures the spike-count variance divided by the mean spike count across 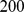 repeated simulations. Spikes were counted in a 50 ms time window and a sliding of 10 ms [18]. As before, we compared our standard adaptive network (**Figure 2G,I**; *τ_s_* = 110 ms) with the control network (**Figure 2H,J**; *τ_s_* = 30 ms). Since the Fano factor is known to be strongly dependent on the firing rate, we adjusted the stimulus level to the latter such that the averaged steady state firing rates in both networks were mean-matched [18]. The input Poisson spike trains (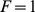) translated into slightly more regular spontaneous (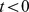) activity in the afferent ensemble (**Figure 2G**), as neuronal membrane filtering and refractoriness reduced the output variability. After the stimulus onset (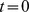), due to the increase in the mean input rate, the average firing rate increased, however the variance of the number of events per trial did not increase proportionally. Therefore, we observed a reduction in the Fano factor (**Figure 2G**). This phenomena is independent of the adaptation mechanism in the neuron model and a quantitatively similar reduction can be observed in the weakly adaptive afferent ensemble (**Figure 2H**). A comparison between our standard adaptive and the control case reveals that the adaptive network is generally more regular in the background and in the evoked state (**Figure 2G,H**). This is due to the previously known effect, where adaptation induces negative serial dependencies in the inter-spike intervals [29], [43] and as a result reduces the Fano factor [29], [44].

In the next stage of processing, the distribution of 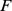 across neurons during spontaneous activity is high due to the self-generated noise of the balanced circuits [23], [24]. This closely follows a wide spread experimental finding where 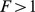 in the spontaneous activity of sensory and motor cortices [18]–[20] (**Figure 2G,H**). This highly variable regime can be achieved in the balance network with strong recurrent couplings [23]. Whenever a sufficiently strong stimulus was applied, the internally generated fluctuations in the adaptive balanced network were transiently suppressed, and as a result the Fano factor dropped sharply (**Figure 2I**). However, this reduction of the Fano factor is a temporary phenomenon and 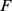 converges back to slightly above the baseline variability (**Figure 2I**). At the same time, the evoked steady-state firing remained in the irregular and asynchronous state (**Figure 2D**). Indeed, this transient effect corresponds to a temporally mismatch in the balanced input conditions to the cortical neurons since the self-inhibitory and slower adaption effect prevents a rapid adjustment to the new input regime. This can be observed in the time course of variability suppression that closely reflects the time constant of adaptation (**Figure 2I**). However, with stronger adaptive feedforward input we can prevent the return of the Fano factor to the base line, this phenomena is due to the regularizing effect of adaptation in the afferent ensemble. In this scenario, the afferent ensemble structured the input to the cortical ensemble, contributing to the magnitude of the observed variability reduction. Indeed, whenever the excitatory feedforward strength is considerably strong, relative to the recurrent connections, the cortical network moves away from the balanced condition. Thus, such strong input resets the internal spiking dynamics within the cortical network and as a result it regulates the spiking variability [45]. This mechanism evidently can be used to prevent the recovery of the high variability. However, we deliberately use a weak stimulation to focus on the transit suppression of cortical variability that is mediated by the slow self-regulation due to adaptation. For instance, under the control condition where a pure Poisson input (with similar synaptic strength) is provided to the cortical balanced network, the reduction in 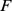 is reduced but the time scale of recovery remains unaltered (crosses in **Figure 2I**). We contrast this adaptive behavior with the variability dynamics in the weakly adaptive balanced network (**Figure 2J**). In this case there is no reduction in 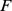, because for a short adaptation time constant the convergence to the balanced state is very rapid [24]. The small increase in the input noise strength leads to an increase of the self-generated randomness of the balanced network [17], [23].

**Figure 3:**
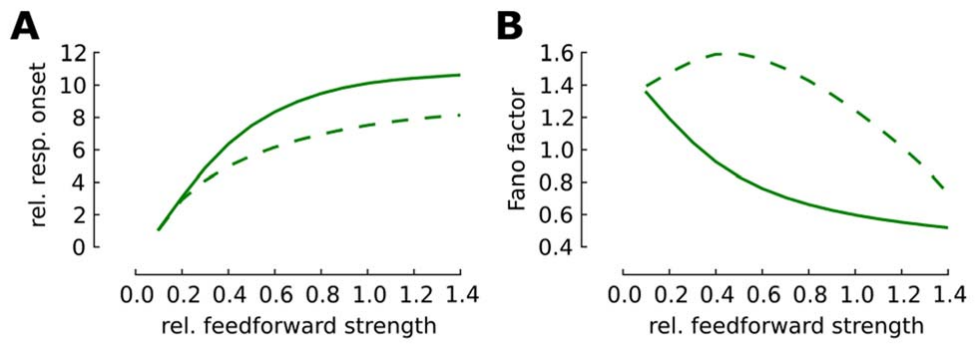
Reliability of a weak and temporally sparse signal in the balanced cortical network. We modify the synaptic strength of the feed-forward input relative to the excitatory recurrent input. The stimulation protocol consists of a brief (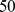
ms) step increase of the Poisson input to the afferent ensemble. (**A**) Amplitude of responses relative to the background firing rate in the cortical layer for adaptive (solid line) and weakly adaptive (dashed line) neurons in dependence of the relative feed-forward coupling strength. (**B**) The Fano factor of the responses given the relative strength of feedforward coupling.

In the above comparison, we adjust the stimulus strength to achieve the same steady-state firing rate (tonic response) in the afferent and cortical ensemble. In a next step we studied the effect of adaptation on the detectability of a weak and transient peripheral signal, which might be impaired by the self-generated noise in the cortical network. To this end we employed the population density approach (**Material and Methods**) to study the mean and variability of the cortical network responses to a wide range of signal strengths. We change the stimulation protocol to a brief signal with a duration of 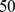
ms over the spontaneous background. The stimulation magnitude is adjusted to elicit the same onset firing rate in the afferent ensemble network in both adaptive and weakly adaptive cases. By modification of the feed-forward coupling between afferent and cortical network relative to the intracortical recurrent coupling we study the circuit responses (**Figure 3A**). Evidently, the strength of the feed-forward coupling to the cortical ensemble modifies its spontaneous background, and therefore also the total adaptation level. The adaptive network proves more sensitive to brief and weak stimuli. It significantly magnifies the mean stimulus response in the adaptive network relative to the background. Even for a considerably weak stimulus the relative amplitude of the response to background firing is pronounced (**Figure 3A**). This result resembles the amplification of a transient in the sequence of adaptive networks as it is observed in the previous section (**Figure 1A**).

How reliable are the responses across trials? To answer this question we calculated the Fano factor (**Material and Methods**) for the cortical ensemble response in the above scenario. This calculation indicates that the response variability in the adaptive network is significantly lower than in the weakly adaptive network over a large range of the feed-forward coupling strength (**Figure 3B**). Interestingly, our results of the population density treatment quantitatively follow the former prediction based on a network simulation that in a balanced network without adaptation the variability initially increases with signal strength (dashed line in **Figure 3B** and Table 1 in [23]) and only after a critical level of the feed-forward strengths the recurrent noise is suppressed due to the stronger influence of excitatory inputs.

### Adaptive networks generate sparse and reliable responses in the insect olfactory system

**Figure 4:**
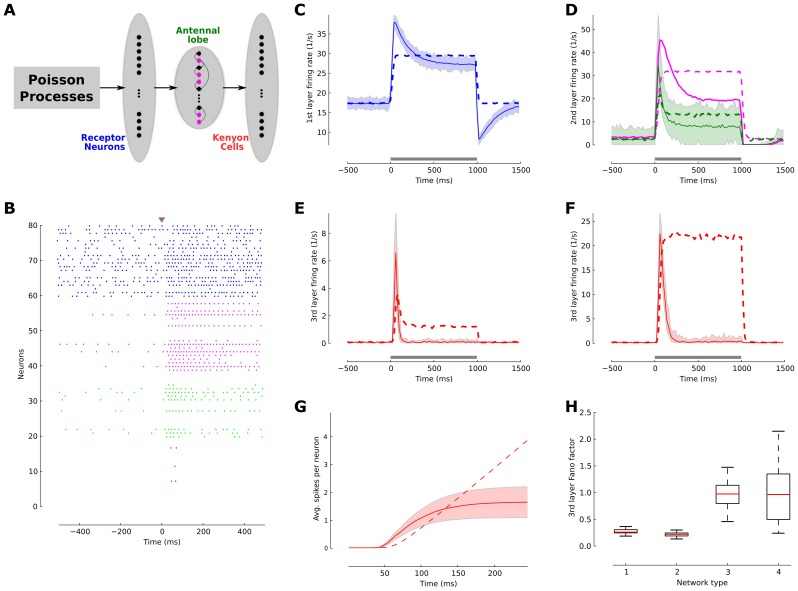
Neuronal adaptation generates temporal sparseness in a generic model of the insect olfactory network. (**A**) Schematic drawing of a simplified model of the insect olfactory network for a single pathway of odor coding. Olfactory receptor neurons (OSNs, first layer, n = 1,480) project to the antennal lobe network (second layer) consisting of projection neurons (PNs, n = 24) and local neurons (magenta, n = 96), which make inhibitory connections with PNs. PNs project to the Kenyon cells (KCs) in the mushroom body (third layer). (**B**) Spike raster plot of randomly selected OSNs (blue), LNs (magenta), PNs (green) and KCs (red) indicates that spiking activity in the network became progressively sparser as the Poisson input propagated into the network. (**C**) Average population rate of OSNs in the adaptive network (blue solid line) and the non-adaptive control network (dashed blue lines). The shaded area indicates the firing rate distribution of the neurons. The firing rate was estimated with 20 ms bin size. (**D**) Average response in the antennal lobe network. PNs (green) and LNs (magenta) exhibited the typical phasic-tonic response profile in the adaptive network (solid lines) but not in the non-adaptive case (dashed lines). (**E**) Kenyon cell activity. In the adaptive network the KC population exhibits a brief response immediately after stimulus onset, which quickly returns close to baseline. This is contrasted by a tonic response profile throughout the stimulus in the non-adaptive case. (**F**) Effect of the inhibitory micro-circuit. By turning off the inhibitory LN-PN connections the population response amplitude of the KCs was increased, while the population response dynamics did not change.(**G**) Sparseness of KCs. The average number of spikes per neuron emitted since stimulus onset indicates that the adaptive ensemble encodes stimulus information with only very few spikes. (**H**) Reliability of KCs responses. The Fano factor of the KCs in different network scenarios is estimated across 200 trials in a 100 ms time window after stimulus onset. Network 1: (+)Adaptation (+)Inhibition, network 2: (+)Adaptation (−)Inhibition, network 3: (−)Adaptation (−)Inhibition, network 4: (−)Adaptation (+)Inhibition. Both networks with SFA are significantly more reliable in their stimulus encoding than the non-adaptive networks.

As a case study to demonstrate how the sequential effect of the adaptation shape responses, we investigated its contribution to the emergence of the reliable and sparse temporal code in the insects olfactory system, which is analogous to the mammalian olfactory system. We simulated a reduced generic model of olfactory processing in insects using the phenomenologically adaptive neuron model [28]. The model network consisted of an input layer with 1,480 olfactory sensory neurons (OSNs), which project to the next layer representing the antennal lobe circuit with 24 projection neurons (PNs) and 96 inhibitory local inter-neurons (LNs) that form a local feed-forward inhibitory micro-circuit with the PNs. The third layer holds 1,000 Kenyon cells (KCs) receiving divergent-convergent input from PNs. The relative numbers for all neurons approximate the anatomical ratios found in the olfactory pathway of the honeybee [46] (**Figure 4A**). We introduced heterogeneity among neurons by randomizing their synaptic time constants and the connectivity probabilities are chosen according to anatomical studies. Synaptic weights were adjusted to achieve spontaneous firing statistics that match the observed physiological regimes. The SFA parameters were identical throughout the network with 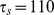
ms (see **Materials and Methods** for details). Experimentally, the cellular mechanisms for SFA exist for neurons at all three network layers [47]–[53]. Notably, strong SFA mediating currents have been identified in the KCs of *Periplaneta americana* [52].

Using this model, we sought to understand how adaptation contributes to temporally sparse odor representations in the KC layer in a small sized network and under highly fluctuating input conditions. We simulated the input to each OSN by an independent Poisson process, which is thought to be reminiscent of the transduction process at the olfactory receptor level [53]. Stimulus activation was modelled by a step increase in the Poisson intensity with uniformly jittered onset across the OSN population (**Materials and Methods**). Following a transient onset response the OSNs adapted their firing to a new steady-state (**Figure 4B,C**). The pronounced effect of adaptation becomes apparent when the adaptive population response is compared to the OSN responses in the control network without any adaptation (*τ_s_* = 0; dashed line, **Figure 4C**). In the next layer, the PN population activity is reflected in a dominant phasic-tonic response profile (green line, **Figure 4D**), which closely matches the experimental observation [54]. This is due to the self-inhibitory effect of the SFA mechanism, and to the feedforward inhibition received from the LNs (magenta line, **Figure 4D**). Consequently, the KCs in the third layer produced only very few action potentials following the response onset with an almost silent background activity (red line, **Figure 4E,G**). The average number of emitted KC response spikes per neuron, 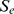, is small in the adaptive network (average 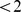) whereas KCs continue spiking throughout stimulus presentation in the non-adaptive network (**Figure 4G**). This finding closely resembles experimental findings of temporal sparseness of KC responses in different insect species [31]–[33] and quantitatively matches the KC response statistics provided by Ito and colleagues [34]. The simulation results obtained here confirm the mathematically derived results in the first results section (**Figure 1B**) and show that neuronal adaption can cause a temporal sparse representation even in a fairly small and highly structured layered network where the mathematical assumptions of infinite network size and fundamentally incoherent activity are not fulfilled (**Materials and Methods**). We further investigated the effect of adaptation on the fraction of responding neurons by counting the number of KCs that emit spikes during stimulation. In the adaptive circuit and in presence of local inhibition only 9% of KCs produce responses (23% in the adaptive network when inhibition is turned off). In contrast, in the non-adaptive network with local inhibition 60% of KCs responded. The low fraction of responding neurons in the adaptive network quantitatively match the experimental findings in the moth [34] and the fruit fly [55].

To test the effect of inhibition in the LN-PN micro-circuitry within the antennal lobe layer on the emergence of temporal sparseness in the KC layer, we deactivated all LN-PN feedforward connections and kept all other parameters fix. We found a profound increase in the amplitude of the KC population response, both in the adaptive (red line, **Figure 4F**) and the non-adaptive network (dashed red line, **Figure 4F**). This increase in response amplitude is carried by an increase in the number of responding KCs due to the increased excitatory input from the PNs, implying a strong reduction in the KC population sparseness. Importantly, removing local inhibition did not alter the temporal profile of the KC population response in the adaptive network (cf. red lines in **Figure 4E,F**), and thus temporal sparseness was independent of inhibition in our network model.

How reliable is the sparse spike response across trials in a single KC? To answer this question, we again measured the robustness of the stimulus representation by estimating the Fano factor across 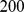 simulation trials (**Figure 4H**). The network with adaptive neurons and inhibitory micro-circuitry exhibited a low Fano factor (median 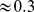) and a narrow distribution across all neurons. This follows the experimental finding that the few spikes emitted by KCs are highly reliably [34] (network 1, **Figure 4H**). Turning off the inhibitory micro-circuitry did not significantly change the response reliability (Wilcoxon rank sum test, p-value = 0.01; network 2, **Figure 4H**). However, both networks that lacked adaptation exhibited a significantly higher variability with a median Fano factor close to one (Wilcoxon rank sum test, p-value = 0.01; networks 3 and 4, **Figure 4H**), independent of the presence or absence of inhibition micro-circuits.

**Figure 5:**
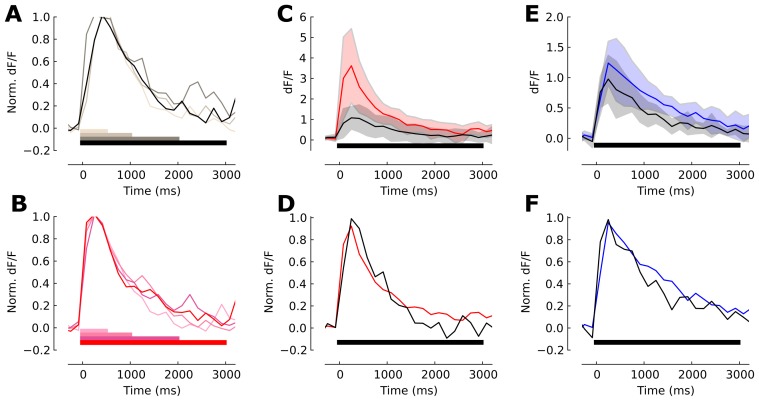
Blocking GABAergic transmission in the honeybee changes amplitude but not duration of the KC population response. (**A**) Temporal response profile of the calcium signal imaged in the mushroom body lip region of one honeybee for different stimulus durations as indicated by color. (**B**) Temporal response profiles as in (**A**) in one honeybee after application of PTX. (**C**) Response profiles imaged from 6 control animals (gray) and their average (black) for a 3 s stimulus as indicated by the stimulus bar. The responses measured in 6 animals in which GABA*_A_* transmission was blocked with PTX (red) shows a considerably higher population response amplitude. The shaded area indicated the standard deviation of responses across bees. (**D**) Average amplitude of responses that are normalized per animal are highly similar in animals treated with PTX and control animals. (**E**) Blocking GABA*_B_* transmission with CGP in 6 animals (blue) again results in an increased response amplitude compared to 6 control animals (black). The shaded area indicates the standard deviation across individuals. (**F**) Average normalized (per animal) response profiles are highly similar in the CGP-treated and control animals.

To explore whether neuronal adaptation could contribute to temporal sparseness in the biological network, we performed a set of Calcium imaging experiments, monitoring Calcium responses in the KC population of the honeybee mushroom body [33] (**Materials and Methods**). Our computational model (**Figure 4**) predicted that blocking of the inhibitory microcircuit would increase the population response amplitude but should not alter the temporal dynamics of the KC population response which is independent of the stimulus duration. In a set of experiments, we tested this hypothesis by comparison of the KCs' evoked activity in the presence and absence of GABAergic inhibition (**Materials and Methods**). First, we analyzed the normalized Calcium response signal within the mushroom body lip region in response to a 3 s, 2 s, 1 s and 0.5 s odor stimulus (**Figure 5A**). We observed the same brief phasic response following stimulus onset in all four cases with a characteristic slope of Calcium response decay that has been reported previously to account for a temporally sparse spiking response [33]. These responses, unlike those of PNs in the previous processing stage of the insect olfactory system [56], [57], are independent of the stimulation duration (**Figure 5A**). Bath application of the GABA*_A_* antagonist picrotoxin (PTX) did not change the time course of the Calcium response dynamics (**Figure 5B–D**). The effectiveness of the drug was verified by the increased population response amplitude in initial phase (**Figure 5C**). Next, we tested the GABA*_B_* antagonist hydrochloride (CGP) using the same protocol and again found an increase in the response magnitude but no alteration of the response dynamics (**Figure 5E,F**). This suggests the absence of inhibition does not change the temporal scale of KCs responses in line with the model prediction.

## Discussion

We propose that a simple neuron-intrinsic mechanism of spike-triggered adaptation can account for a reliable and temporally sparse sensory stimulus representation across stages of sensory processing. The emergence of a sparse representation has been demonstrated in various sensory areas, for example in visual [58], auditory [59], somatosensory [60], and olfactory [61] cortices, and thus manifests a principle of sensory computation across sensory modalities and independent of the natural stimulus kinetic. Our results show that adaptation allows to reliably represent a stimulus with a temporally restricted response to stimulus onset and thus more stimuli can be represented in time which is the basis for a temporally sparse representations of a dynamically changing stimulus environment.

At the single neuron level, SFA is known to induce the functional property of a fractional differentiation with respect to the temporal profile of the input and thus offers the possibility of tuning the neuron's response properties to the relevant stimulus time-scales at the cellular level [2]–[4], [62]–[65]. Our results indicate further that sensory processing in a feedforeward network with adaptive neurons focuses on the temporal changes of the sensory input in a precise and temporally sparse manner (**Figure 1B** **;** **Figure 2E** **and** **Figure 4E**) and at the same time the constancy of the stimulus is memorized in the cellular level of adaptation [37] (**Figure 1D**). The constancy of the environment is an important factor of state-dependent computations [66] that evidently should be tracked by the network. Such context-dependent modulations set the background and have been observed in different sensory systems where responses are strongly influenced by efferent contextual input [67]–[69]. In this paper, we show that information about the context of a given stimuli maybe stored in the adaptation level across processing stages while at the same time the network remains sensitive to changes. Thus, sequential adaptive populations adjust the circuit transfer function in a self-organizing manner to avoid response attenuation to secondary stimuli. These results add a further possibility of network level interactions to the previous suggestions that SFA optimizes the context depended responses and resolves ambiguity in the neuronal code [8], [70] at the single neuron level. This allows a sensory system to detect extremely small changes in stimulus over a large background by means of an adaptive response without contextual information loss [39]. One prominent example is primate vision where, in the absence of the self-generated dynamics of retina input due to microsaccades, observers become functionally blind to stationary objects during fixations [71].

A sparse temporal representation of stimulus permits very few spikes to transmit high quantities of information about a behaviorally significant stimulus [72]. However, it has been repeatedly questioned whether a few informative spikes can survive in the cortical network, which is highly sensitive to small perturbations [16], [73]. Our results show that a biologically realistic cellular mechanism implemented at successive network stages can transform a dense and highly variable Poisson input at the periphery into a temporally sparse and highly reliable ensemble representation in the cortical network. Therefore, it facilitates a transition from a rate code to a temporal code as required for the concerted spiking of cortical cell assemblies [74] (**Figure 2D**). These results reflect previous theoretical evidence that SFA has an extensive synchronizing-desynchronizing effect on population responses in a feedback coupled network [75], [76].

A balance between excitation and inhibition leads to strong temporal fluctuations and produce spike trains with high variability in cortex [16], [23]–[25], [77]. However, the adaption level adjusts with a dynamics that is slow compared to the dynamics of excitatory and inhibitory synaptic inputs. This circumstance allows for a transient mismatch of the balanced state in the cortical network and thus leads to a transient reduction of the self-generated (recurrent) noise (**Figure 2I**). This, in turn, explains why the temporally sparse representation can be highly reliable, following the experimental observations [21], [22]. Moreover, a recent and highly relevant *in vivo* data set hints toward our theoretical prediction, where adaptation may alter the balance between excitation and inhibition and increase the sensitivity of cortical neurons to sensory stimulation [78]. Here, our main result exploited the transient role of adaptation mechanisms on the cortical variability suppression, after which the variability recovers to the unstimulated values, even though the network remains stimulated (**Figure 2I**). One can achieve a longer time scale of variability suppression by an increase in the effect of the afferent strength (as a network mechanism), due to the reduction of the input irregularity in the evoked state. This proposal can be supported by the experimental evidence that thalamic inputs strongly drive neurons in cortex [79] and fits the previous theoretical suggestion by [45]. Noteworthy, in our model the irregularity of inter-spike intervals, measured by 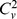, in the balanced network does not change significantly in different conditions, which matches the experimentally reported evidence [80]. The recent theoretical studies [26], [27] show that the slow time scales variability suppression can be also achieved within a clustered topology in the balanced network [26] or likewise in an attractor-based networks of cortical dynamics [27]. In these approaches, the reduced variability can be attributed to an increased regularity of the spike trains. This hints that further research to understand the role of interactions between the network and cellular mechanisms in the cortical variability and other network statistics are certainly needed. Additionally, the link between the temporal sparseness achieved here by cascaded network of adaptive neurons with spatial sparseness of responses [81], [82] requires more elaborated research.

The insect olfactory system is experimentally well investigated and exemplifies a pronounced sparse temporal coding scheme at the level of the mushroom body KCs. The olfactory system is analog in invertebrates and vertebrates and the sparse stimulus representation is likewise observed in the pyramidal cells of the piriform cortex [61], and the rapid responses in the mitral cells in the olfactory bulb [83] compare to those of projection neurons in antennal lobe [54], [84]. Our adaptive network model, designed in coarse analogy to the insect olfactory system, produced increasingly phasic population responses as the stimulus-driven activity propagated through the network. Our model results closely match the repeated experimental observation of temporally sparse and reliable KC responses in extracellular recordings from the locust [31], fruit fly [85] and manduca [32], [34], and in Calcium imaging in the honeybee [33]. Although Calcium responses are slow, it has been suggested that they closely correspond to the population activity dynamics [86]. In our experiments we could show that systemic blocking of GABAergic transmission did not affect the temporal sparseness of the KC population response in the honeybee (**Figure 5**) signified by the transient Calcium response [33]. Therefore, the stable temporal activity in the mushroom body qualitatively matches with our theoretical predication of population rate dynamics (**Figure 1**). This result might seem to contradict former studies that stressed the role of inhibitory feed-forward [87] or feedback inhibition [88], [89] for the emergence of KC sparseness. However, the suggested inhibitory mechanisms and the sequential effect in the adaptive network proposed here are not mutually exclusive and may act in concert to establish and maintain a temporal and spatial sparse code in a rich and dynamic natural olfactory scene. In this paper, we deliberately focus on the temporal aspect of the responses, since it seems that spatial sparseness is mediated by connectivity schemes [90]–[92].

The adaptive network model manifests a low trial-to-trial variability of the sparse KC responses that typically consist of only 1–2 spikes. In consequence, a sparsely activated KC ensemble is able to robustly encode stimulus information. The low variability at the single cell level (**Figure 4H**) carries over to a low variability of the population response [29], [30]. This benefits downstream processing in the mushroom body output neurons that integrate converging input from many KCs [46], and which were shown to reliably encode odor-reward associations in the honeybee [93].

Next to the cellular mechanism of adaptation studied here, short-term synaptic plasticity may produce similar effects. The activity-dependent nature of short term depression (STD) produces correlated presynaptic input spike trains [94]. Hence, it facilitates weak signal detection [94] similar to adaptation [95]. Moreover, STD can also generate a sharp transient in the stimulus response [96], [97] that can propagate to higher layers of the network. Therefore it is plausible to utilize short-term synaptic plasticity to achieve similar results to the ones obtained here with SFA. However, STD may have some drawbacks in comparison to adaptation, namely a low signal-to-noise ratio, and a low-pass filtering of input that is more sensitive to high frequency synaptic noise [62], [68]. Evidently, STD takes effect at the single synapse while SFA acts on a neuron's output. The combination of both mechanisms that are encountered side-by-side in cortical circuits [99], [100] may provide a powerful means for efficient coding [98].

Our results here are of general importance for sensory coding theories. A mechanism of self-inhibition at the cellular level can facilitate a temporally sparse ensemble code but does not require well adjusted interplay between excitatory and inhibitory circuitry at the network level. This network effect is robust due to the distributed nature of the underlying mechanism, which acts independently in each single neuron. The regularizing effect of self-inhibition increases the signal-to-noise ratio not only of single neuron responses but also of the neuronal population activity [29], [30], [37] that is post-synaptically integrated in downstream neurons.

## Materials and Methods

### Rate model of a generic feedforward adaptive network

**Figure 6:**
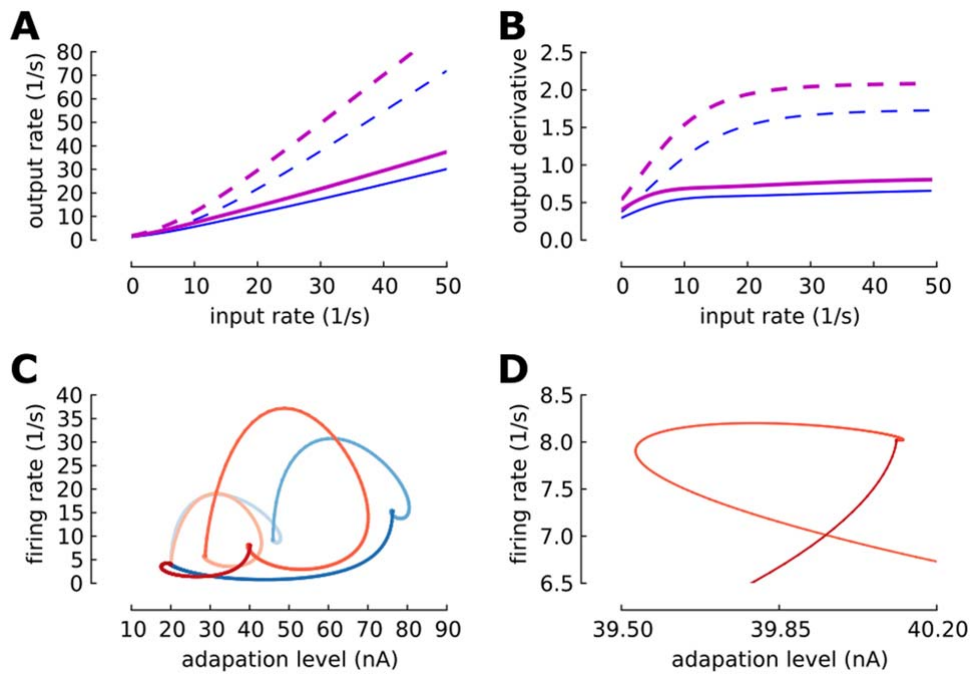
Response properties of the rate model. (**A**) The input-output transfer function of a population, where the adaptation is ineffective or not yet adjust with the input (dashed line) and at the adapted steady-state (solid line). During the transient response the dashed line is a good approximation for the adaptive population. The magenta lines indicate the case where the coupling strength is 20% increased compared to the blue lines (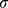 = 1000[nA], 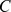 = 500[nF], 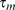 = 20[ms], 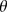 = 20[mV],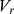 = 0[mV], 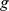 = 20 [ms][nA] and 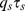 = 5 [ms][nA]). (**B**) The derivative of the response functions given in (A) with respect to the input rate. (**C**) The adaptation-rate phase plot of the first (blue) and third layer (red) in **Figure 1D** with a two-step stimulus input. The time is encoded in the contrast of the lines (the lowest contrast is 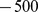
ms and the highest is 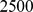
ms). The system exhibits a stable focus with the under shoot during the relaxation to baseline. (**D**) Zoom in of (C) showing the convergence to the adapted stated for the second step increase of the stimulus in the third layer (low contrast line) and the relaxation to the base line (high contrast line).

To address analytically the sequential effect of adaptation in a feedforward network, we consider a model in which populations are described by their firing rates. Although firing rate models typically provide a fairly accurate description of network behavior when the neurons are firing asynchronously [101], they do not capture all features of realistic networks. Therefore, we verify all of our predictions with a population density formalism [29] as well as a large-scale simulation of realistic spiking neurons. To determine the mean activity dynamics of a consecutive populations, we employed an standard mean firing rate model of population 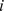 as
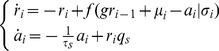
(1)where 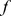 is the transfer input-output function, 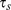 is the adaptation time-scale, 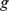 is the coupling factor between two populations and 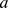 is the adaptive negative feedback for the population 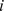 with 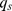 strength and 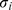 is the standard deviation of the input. In our rate model analysis, we use the transfer function of the leaky-integrate and fire neuron that can be written as
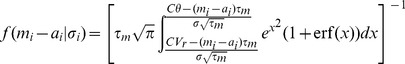
(2)where 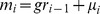 and 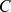, 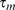, 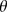 and 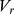 are membrane capacitance, membrane time-constant, spiking threshold and reset potential, respectively. Here, we assume 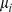 is the injected current to the population 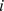 independent of the stimulus and constant over time. Given 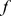, the equilibrium can be determined by
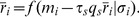
(3)The condition for the stability reads 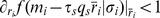 and 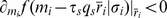 [35] (**Figure 6A**). It is important to note that whenever the conditions for stability are satisfied, the fix point is reached via a *focus* attractor (**Figure 6 C,D**) since the Jacobin of this system (under the physiological condition of 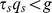) always has a complex eigenvalue with a negative real part. It is also known that the 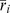 is a linear function with respect to its input, given a sufficiently slow 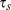 or strong adaptation 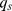 and a non-linear shape of 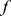 [35], [36], [102]. It can also be shown that whenever the adaptation is ineffective (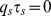) we have
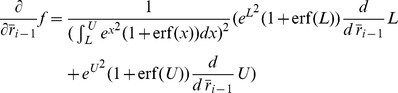
(4)where 
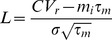
 and 
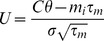
. This derivative scales with 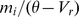 (**Figure 6B**). Now, we can plug back the adaptation into the steady state solution, which has a magnitude of 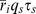. In **Figure 6 B**, we numerically determine the condition for 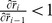, that it reads 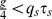, given the parameters 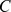, 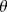, 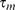, 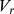 and 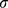 as they are stated in the caption. An increase in the population rate 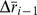 leads to a reduced increase 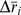 in the next population, and therefore the adapted level of responses satisfy 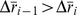. For realistic adaptation values this mapping closely follows the result of a previous study where it was shown that the effect of increasing the cells input conductance on its f-I curve is mainly subtractive [102]. Note that for very weak adaptation the steady-state is not affected considerably.

The magnitude of the transient response firing rate for an adaptive population lies between the adapted steady-state rate and the response rate without adaptation. Given the level of new input it can be calculated analytically [2]. Hence, the slow dynamics of adaptation and fast response f-I curve reflect two states of operation where the onset response very closely follows the properties of the non-adapted response curve and the adapted steady state produces a subtractive input-output relationship. Noteworthy, the assumption that all populations have a same background firing rate is not a necessary condition. One can achieve the same result by using heterogeneous couplings 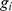 or stimulus independent private input 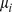 that may induce more realistic variations in background rates as observed in different stages of sensory processing. The crucial point of the inherited dynamics due to adaption is the fundamental non-linearity that (1) the transient response amplitude is hardly affected by adaptation, (2) the adapted steady-state is fall apart from it and can become subtractive.

### Population density approach to the adaptive neuronal ensemble

In Muller et al. [28] it is shown that by an adiabatic elimination of fast variables a detailed neuron model including voltage dynamics, conductance-based synapses, and spike-induced adaptation, in the incoherent state (Asynchronous and Irregular state) reduces to a stochastic point process. Thus, we define an *orderly point process* with a hazard function argument with state variable 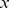 as
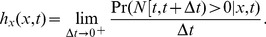
(5)where 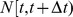 is the number of events in 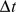. We assume the dynamic of the adaptation variable is
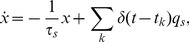
(6)where 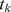 is the time of 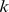
th spike in the ensemble. Thus, the state variable distribution at time 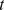 in the ensemble is governed by a master equation of the form
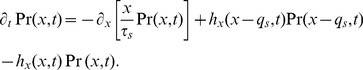
(7)We solve Eq. 7 with the help of the transformations 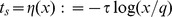 and 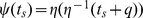 numerically [28]. The master equation here belongs to a non-renewal process [28], [29] and its renewal correspondence can be seen in [28]. It turns out that indeed 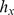 is the input-out transfer function of neurons in the network where its instantaneous parameters are give by the input statistics [28]. For instance, the transfer function of a conductance based leaky integrate and fire neuron can be written as
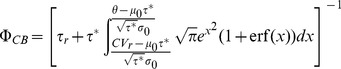
(8)where, 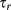 and 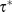 are refectory period and an input-dependent effective time constants, respectively. The 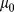 and 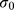 appearing in 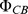 are the average and variance of the free (i.e., spike-less) membrane voltage distribution [35] and 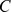 is the membrane capacitance. Here, we used the mean-field formalism developed by [23] and [103] to approximately determine the averaged input within a standard balanced network, as the parameters of the hazard function 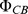 suggested by [35], which uses the calculated average firing rate of inhibitory 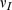 and excitatory neurons 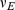 in the randomly connected network. The analytical results in this paper assumed the standard 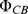 as a from of the hazard function 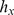 and the value of 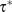 is estimated form simulations of the detailed neuron model with an step like input increase. Here, it is important to note that conductance-based model approximately follows the current based neuron model with a colored noise, where 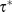 is shorter than the membrane time constant of the neuron, which now depends on the total conductance [103], [104]. Therefore, given the 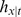 estimate, we can approximate the numerical solution of the master equation (eq. 7) by applying the exponential Euler method for the death term, and reinserting the lost probability as it is fully described in [28]. The functional form of the solution in a compact form can be written as
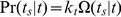
(9)where 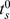 is the initial condition state of the system and 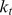 is a constant defined by 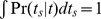 [28], [29]. Similarly, one can derive the distribution of 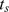 just after the event, 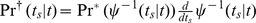 [28]. Then, the relationship between 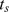 and the ordinary ISI distribution can be written as
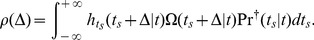
(10)where 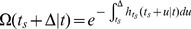. Now the 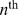 moment 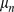 of the distribution and its coefficient of variation 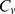 can be numerically determined. Note that the framework here is closely connected to the spike response model, also known as the generalized linear neuron model [30]. Alternatively, the same ISI distribution can be also derived form the discretization of the master equation as it is demonstrated in [37]. It can be shown that the firing rate and the consistency equation of the ensemble is
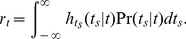
(11)Now to calculate the counting statistics, we applied the techniques are introduced by Farkhooi et al [29], and defined a joint probability density as
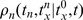
(12)where an 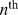 event occurs at time 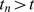 and the state variable is 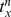. We can write 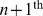 event time and state of adaptation joint density recursively,
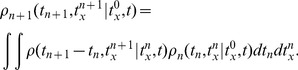
(13)To simplify the integral equations, we use Bra-Ket notation following a suggestion by [105], defined as
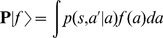
(14)and
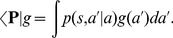
(15)Thereafter, we derive the Laplace transform (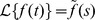) of the joint density in the eq. 13 by
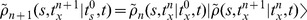
(16)where 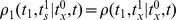. Next, we define the operator 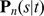,
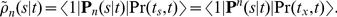
(17)Now, by employing 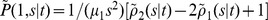 as in [29], we derive
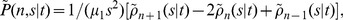
(18)where 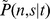 is the Laplace transform of the probability density of observing 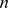 events in a given time window. Now we derive
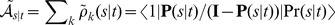
(19)where 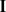 is the identity operator. This equation represents the Laplace transform of the auto-correlation function. Using auto-correlation function 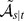, we can calculate the Fano factor that provides an index for the quantification of the count variability. It is defined as 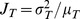, where 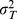 and 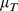 are the variance and the mean of the number of events in a certain time window 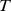. It follows from the additive property of the expectation that 
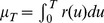
 and in the case of a constant firing rate, it is simply 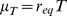. To calculate the second moment of 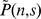, we require 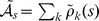 in eq. 19. Thus, the Fano factor is 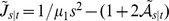 and the inverse Laplace transform is
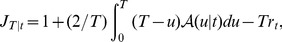
(20)where 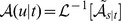. In [29], we demonstrate in detail that the asymptotic property of 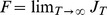 at equilibrium can be derived as
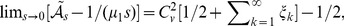
(21)where 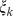 is the linear correlation coefficient between two 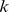 lagged intervals. Provided the limit exits, we find the familiar relationship of 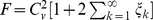 in the steady-state.

### Computational model of insect olfactory network

Our model neuron is a general conductance-based integrate-and-fire neuron with spike-frequency adaptation as it is proposed in Muller et al. [28]. The model phenomenologically captures a wide array of biophysical spike-frequency mechanisms such as M-type current, afterhyperpolarization (AHP-current) and even slow recovery from inactivation of the fast sodium current [28]. The model neuron is also known to perform high-pass filtering of the input frequencies following the universal model of adaptation [2]. Neuron parameters used follow the Table 3 in Muller et al. [28]. The conductance model used for the static synapses between the neurons is alpha-shaped with gamma distributed time constants from 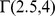 and 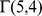 for excitatory and inhibitory synapses, respectively. All simulations were performed using the NEST simulator [106] version 2.0beta and the Pynest interface.

The network connectivity is straight forward: each PN and LN receives excitatory connections from 20% randomly chosen OSNs [57], [107]. Additionally, every PN receives input from 50% randomly chosen inhibitory LNs [57], [107]. In our model the PNs do not excite one another and each PN output diverges to 50% randomly chosen KCs [33], [87], [90].

We tuned the simulated network by adjusting the synaptic weights to achieve the same spontaneous firing rate as reported experimentally: OSNs 15–25 Hz [53], LNs 4–10 Hz [107], PNs 3–10 [54] and KCs 0.3–1.0 Hz [34].

### Experimental methods

Experiments were performed following the methods published in Szyszka et al [33]. In summary, foraging honeybees (*Apis mellifera*) were caught at the entrance of the hive, immobilized by chilling on ice, and fixed in a plexiglas chamber before the head capsule was opened for dye injection. We retrogradely stained clawed Kenyon cells (KC) of the median calyx, using the calcium sensor FURA-2 dextran (Molecular Probes, Eugene, USA) with a dye loaded glass electrode, which was pricked into KC axons projecting to the ventral median part of the 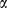-lobe [33]. After dye injection the head capsule was closed, bees feed and kept in a dark humid chamber for several hours.

The processing of imaging data was performed with custom written routines in IDL (RSI, Boulder, CO, USA). In summary, changes in the calcium concentration were measured as absolute changes of fluorescence: a ratio was calculated from the light intensities measured at 340 nm and 380 nm illumination and the background fluorescence before odor onset was subtracted leading to 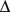
F with F = F340/F380. Odor stimulation was preformed under a 20× objective of the microscope, the naturally occurring plant odor octanol (Sigma Aldrich, Germany), diluted 1:100 in paraffine oil (FLUKA, Buchs, Switzerland), was delivered to both antennae of the bee using a computer controlled, custom made olfactometer. To this, odor loaded air was injected into a permanent airstream resulting in a further 1:10 dilution. Stimulus duration was 3 seconds if not mentioned otherwise. The air was permanently exhausted.

For GABA blockage, a solution of 150 µl GABA receptor antagonist dissolved in ringer for final concentration (10^−5^M picrotoxin (PTX, Sigma Aldrich, Germany) or 5×10^−4^M CGP54626 (CGP, Tocris Bioscience, USA)) was bath applied to the brain after pre-treatment measurements. Measurements started 10 min after drug application. The calcium signals are analyzed in Matlab (The MathWorks Inc., Natick, USA). The normalization of the responses are performed per animal and the plotted traces are the averaged values across subjects.

## References

[1] Adrian ED (1926) The impulses produced by sensory nerve endings. The Journal of physiology 61: 4972. 10.1113/jphysiol.1926.sp002273 PMC1514809 16993776

[2] Benda J, Herz AVM (2003) A universal model for spike-frequency adaptation. Neural Computation 15: 2523–2564. 14577853 10.1162/089976603322385063

[3] Lundstrom BN, Higgs MH, Spain WJ, Fairhall AL (2008) Fractional differentiation by neocortical pyramidal neurons. Nature Neuroscience 11: 1335–1342. 18931665 10.1038/nn.2212 PMC2596753

[4] Thorson J, Biederman-Thorson M (1974) Distributed relaxation processes in sensory adaptation spatial nonuniformity in receptors can explain both the curious dynamics and logarithmic statics of adaptation. Science 183: 161–172. 4587440 10.1126/science.183.4121.161

[5] Rudy B (1988) Diversity and ubiquity of k channels. Neuroscience 25: 729–749. 2457185 10.1016/0306-4522(88)90033-4

[6] Ranganathan R (1994) Evolutionary origins of ion channels. Proceedings of the National Academy of Sciences of the United States of America 91: 3484. 7513423 10.1073/pnas.91.9.3484 PMC43604

[7] Koshland D Jr (1983) The bacterium as a model neuron. Trends in Neurosciences 6: 133–137.

[8] Wark B, Lundstrom BN, Fairhall A (2007) Sensory adaptation. Current opinion in neurobiology 17: 423–429. 17714934 10.1016/j.conb.2007.07.001 PMC2084204

[9] Shapley R, Enroth-Cugell C (1984) Visual adaptation and retinal gain controls. Progress in retinal research 3: 263–346.

[10] Laughlin SB (1989) The role of sensory adaptation in the retina. Journal of Experimental Biology 146: 3962. 10.1242/jeb.146.1.39 2689569

[11] Laughlin SB, Hardie RC (1978) Common strategies for light adaptation in the peripheral visual systems of y and dragony. Journal of comparative physiology 128: 319–340.

[12] Hecht S, Shlaer S, Pirenne M (1942) Energy, quanta, and vision. The Journal of general physiology 25: 819840. 10.1085/jgp.25.6.819 PMC2142545 19873316

[13] Faisal AA, Selen LPJ, Wolpert DM (2008) Noise in the nervous system. Nature reviews Neuroscience 9: 292–303. 18319728 10.1038/nrn2258 PMC2631351

[14] Barlow HB (1969) Trigger features, adaptation and economy of impulses. Information Processing in the Nervous System 209230.

[15] Stein RB, Gossen ER, Jones KE (2005) Neuronal variability: noise or part of the signal? Nat Rev Neurosci 6: 389–397. 15861181 10.1038/nrn1668

[16] London M, Roth A, Beeren L, Hausser M, Latham PE (2010) Sensitivity to perturbations in vivo implies high noise and suggests rate coding in cortex. Nature 466: 123–127. 20596024 10.1038/nature09086 PMC2898896

[17] Monteforte M, Wolf F (2010) Dynamical entropy production in spiking neuron networks in the balanced state. Physical Review Letters 105: 268104. 21231716 10.1103/PhysRevLett.105.268104

[18] Churchland MM, Yu BM, Cunningham JP, Sugrue LP, Cohen MR, et al (2010) Stimulus onset quenches neural variability: a widespread cortical phenomenon. Nat Neurosci 13: 369–378. 20173745 10.1038/nn.2501 PMC2828350

[19] Churchland MM, Yu BM, Ryu SI, Santhanam G, Shenoy KV (2006) Neural variability in premotor cortex provides a signature of motor preparation. The Journal of Neuroscience 26: 3697–3712. 16597724 10.1523/JNEUROSCI.3762-05.2006 PMC6674116

[20] Nawrot MP, Boucsein C, Rodriguez Molina V, Riehle A, Aertsen A, et al (2008) Measurement of variability dynamics in cortical spike trains. Journal of Neuroscience Methods 169: 374–390. 18155774 10.1016/j.jneumeth.2007.10.013

[21] Herikstad R, Baker J, Lachaux JP, Gray CM, Yen SC (2011) Natural movies evoke spike trains with low spike time variability in cat primary visual cortex. The Journal of Neuroscience 31: 15844–15860. 22049428 10.1523/JNEUROSCI.5153-10.2011 PMC6623039

[22] Haider B, Krause MR, Duque A, Yu Y, Touryan J, et al (2010) Synaptic and network mechanisms of sparse and reliable visual cortical activity during nonclassical receptive field stimulation. Neuron 65: 107–121. 20152117 10.1016/j.neuron.2009.12.005 PMC3110675

[23] Lerchner A, Ursta C, Hertz J, Ahmadi M, Ruffot P, et al (2006) Response variability in balanced cortical networks. Neural Computation 18: 634–659. 16483411 10.1162/089976606775623261

[24] van Vreeswijk C, Sompolinsky H (1998) Chaotic balanced state in a model of cortical circuits. Neural Comput 10: 1321–71. 9698348 10.1162/089976698300017214

[25] Renart A, de la Rocha J, Bartho P, Hollender L, Parga N, et al (2010) The asynchronous state in cortical circuits. Science 327: 587–590. 20110507 10.1126/science.1179850 PMC2861483

[26] Litwin-Kumar A, Doiron B (2012) Slow dynamics and high variability in balanced cortical networks with clustered connections. Nature Neuroscience 15: 1498–1505. 23001062 10.1038/nn.3220 PMC4106684

[27] Deco G, Hugues E (2012) Neural network mechanisms underlying stimulus driven variability reduction. PLoS Comput Biol 8: e1002395. 22479168 10.1371/journal.pcbi.1002395 PMC3315452

[28] Muller E, Buesing L, Schemmel J, Meier K (2007) Spike-frequency adapting neural ensembles: Beyond mean adaptation and renewal theories. Neural Comp 19: 2958–3010. 10.1162/neco.2007.19.11.2958 17883347

[29] Farkhooi F, Muller E, Nawrot MP (2011) Adaptation reduces variability of the neuronal population code. Physical Review E 83: 050905. 10.1103/PhysRevE.83.050905 21728481

[30] Naud R, Gerstner W (2012) Coding and decoding with adapting neurons: A population approach to the peri-stimulus time histogram. PLoS Comput Biol 8: e1002711. 23055914 10.1371/journal.pcbi.1002711 PMC3464223

[31] Perez-Orive J, Mazor O, Turner GC, Cassenaer S, Wilson RI, et al (2002) Oscillations and sparsening of odor representations in the mushroom body. Science 297: 359–65. 12130775 10.1126/science.1070502

[32] Broome BM, Jayaraman V, Laurent G (2006) Encoding and decoding of overlapping odor sequences. Neuron 51: 467–82. 16908412 10.1016/j.neuron.2006.07.018

[33] Szyszka P, Ditzen M, Galkin A, Galizia CG, Menzel R (2005) Sparsening and temporal sharpening of olfactory representations in the honeybee mushroom bodies. Journal of Neurophysiology 94: 3303–3313. 16014792 10.1152/jn.00397.2005

[34] Ito I, Ong RCy, Raman B, Stopfer M (2008) Sparse odor representation and olfactory learning. Nature Neuroscience 11: 1177–1184. 18794840 10.1038/nn.2192 PMC3124899

[35] LaCamera G, Rauch A, Luscher HR, Senn W, Fusi S (2004) Minimal models of adapted neuronal response to in vivo-like input currents. Neural Comput 16: 21012124. 10.1162/0899766041732468 15333209

[36] Ermentrout B (1998) Linearization of FI curves by adaptation. Neural computation 10: 17211729. 10.1162/089976698300017106 9744894

[37] Nesse WH, Maler L, Longtin A (2010) Biophysical information representation in temporally correlated spike trains. Proceedings of the National Academy of Sciences 107: 21973–21978. 10.1073/pnas.1008587107 PMC3009835 21131567

[38] Kadohisa M, Wilson DA (2006) Olfactory cortical adaptation facilitates detection of odors against background. Journal of Neurophysiology 95: 1888–1896. 16251260 10.1152/jn.00812.2005 PMC2292127

[39] Koshland DE, Goldbeter A, Stock JB (1982) Amplification and adaptation in regulatory and sensory systems. Science 217: 220–225. 7089556 10.1126/science.7089556

[40] Kumar A, Schrader S, Aertsen A, Rotter S (2008) The high-conductance state of cortical networks. Neural Computation 20: 143. 10.1162/neco.2008.20.1.1 18044999

[41] Vogels TP, Abbott LF (2009) Gating multiple signals through detailed balance of excitation and inhibition in spiking networks. Nat Neurosci 12: 483–491. 19305402 10.1038/nn.2276 PMC2693069

[42] Roxin A, Brunel N, Hansel D, Mongillo G, van Vreeswijk C (2011) On the distribution of firing rates in networks of cortical neurons. The Journal of Neuroscience 31: 16217–16226. 22072673 10.1523/JNEUROSCI.1677-11.2011 PMC6633220

[43] Benda J, Maler L, Longtin A (2010) Linear versus nonlinear signal transmission in neuron models with adaptation currents or dynamic thresholds. Journal of Neurophysiology 104: 2806–2820. 21045213 10.1152/jn.00240.2010

[44] Chacron MJ, Maler L, Bastian J (2005) Electroreceptor neuron dynamics shape information transmission. Nature Neuroscience 8: 673–678. 15806098 10.1038/nn1433 PMC5283878

[45] Rajan K, Abbott L, Sompolinsky H (2010) Stimulus-dependent suppression of chaos in recurrent neural networks. Physical Review E 82: 011903. 10.1103/PhysRevE.82.011903 PMC10683875 20866644

[46] Menzel R, Squire LR (2009) Olfaction in invertebrates: Honeybee. In: Encyclopedia of Neuroscience. Oxford: Academic Press. pp. 43–48.

[47] Kaissling K, Strausfeld CZ, Rumbo ER (1987) Adaptation processes in insect olfactory receptors. Annals of the New York Academy of Sciences 510: 104–112. 3324874 10.1111/j.1749-6632.1987.tb43475.x

[48] Mercer AR, Hildebrand JG (2002) Developmental changes in the density of ionic currents in Antennal-Lobe neurons of the sphinx moth, manduca sexta. J Neurophysiol 87: 2664–2675. 12037169 10.1152/jn.2002.87.6.2664

[49] Grunewald B (2003) Differential expression of voltage-sensitive k+ and ca2+ currents in neurons of the honeybee olfactory pathway. J Exp Biol 206: 117–129. 12456702 10.1242/jeb.00053

[50] Wüstenberg DG, Boytcheva M, Grünewald B, Byrne JH, Menzel R, et al (2004) Current- and Voltage-Clamp recordings and computer simulations of kenyon cells in the honeybee. Journal of Neurophysiology 92: 2589–2603. 15190098 10.1152/jn.01259.2003

[51] Schafer S, Rosenboom H, Menzel R (1994) Ionic currents of kenyon cells from the mushroom body of the honeybee. J Neurosci 14: 4600–4612. 7519255 10.1523/JNEUROSCI.14-08-04600.1994 PMC6577176

[52] Demmer H, Kloppenburg P (2009) Intrinsic membrane properties and inhibitory synaptic input of kenyon cells as mechanisms for sparse coding? J Neurophysiol 102: 1538–1550. 19553491 10.1152/jn.00183.2009

[53] Nagel KI, Wilson RI (2011) Biophysical mechanisms underlying olfactory receptor neuron dynamics. Nature Neuroscience 14: 208–216. 21217763 10.1038/nn.2725 PMC3030680

[54] Krofczik S, Menzel R, Nawrot MP (2008) Rapid odor processing in the honeybee antennal lobe network. Frontiers in Computational Neuroscience 2: 9. 19221584 10.3389/neuro.10.009.2008 PMC2636688

[55] Honegger KS, Campbell RAA, Turner GC (2011) Cellular-resolution population imaging reveals robust sparse coding in the drosophila mushroom body. The Journal of Neuroscience 31: 11772–11785. 21849538 10.1523/JNEUROSCI.1099-11.2011 PMC3180869

[56] Sachse S, Galizia CG (2003) The coding of odour-intensity in the honeybee antennal lobe: local computation optimizes odour representation. European Journal of Neuroscience 18: 21192132. 10.1046/j.1460-9568.2003.02931.x 14622173

[57] Sachse S, Galizia CG (2002) Role of inhibition for temporal and spatial odor representation in olfactory output neurons: A calcium imaging study. J Neurophysiol 87: 1106–1117. 11826074 10.1152/jn.00325.2001

[58] Tolhurst DJ, Smyth D, Thompson ID (2009) The sparseness of neuronal responses in ferret primary visual cortex. The Journal of Neuroscience 29: 2355–2370. 19244512 10.1523/JNEUROSCI.3869-08.2009 PMC6666250

[59] Hromdka T, DeWeese MR, Zador AM (2008) Sparse representation of sounds in the unanesthetized auditory cortex. PLoS Biol 6: e16. 18232737 10.1371/journal.pbio.0060016 PMC2214813

[60] Jadhav SP, Wolfe J, Feldman DE (2009) Sparse temporal coding of elementary tactile features during active whisker sensation. Nature Neuroscience 12: 792–800. 19430473 10.1038/nn.2328

[61] Poo C, Isaacson JS (2009) Odor representations in olfactory cortex: Sparse coding, global inhibition, and oscillations. Neuron 62: 850–861. 19555653 10.1016/j.neuron.2009.05.022 PMC2702531

[62] Tripp B, Eliasmith C (2009) Population models of temporal differentiation. Neural Computation 22 (3) 621–59. 10.1162/neco.2009.02-09-970 19922294

[63] Benda J, Longtin A, Maler L (2005) Spike-frequency adaptation separates transient communication signals from background oscillations. J Neurosci 25: 2312–2321. 15745957 10.1523/JNEUROSCI.4795-04.2005 PMC6726095

[64] Ulanovsky N, Las L, Farkas D, Nelken I (2004) Multiple time scales of adaptation in auditory cortex neurons. J Neurosci 24: 10440–10453. 15548659 10.1523/JNEUROSCI.1905-04.2004 PMC6730303

[65] Brenner N, Bialek W, de Ruyter van Steveninck R (2000) Adaptive rescaling maximizes information transmission. NEURON-CAMBRIDGE MA- 26: 695702. 10.1016/s0896-6273(00)81205-2 10896164

[66] Buonomano DV, Maass W (2009) State-dependent computations: spatiotemporal processing in cortical networks. Nature Reviews Neuroscience 10: 113–125. 19145235 10.1038/nrn2558

[67] Kay LM, Laurent G (1999) Odor- and context-dependent modulation of mitral cell activity in behaving rats. Nature Neuroscience 2: 1003–1009. 10526340 10.1038/14801

[68] Malone BJ, Scott BH, Semple MN (2002) Context-dependent adaptive coding of interaural phase disparity in the auditory cortex of awake macaques. The Journal of Neuroscience 22: 4625–4638. 12040069 10.1523/JNEUROSCI.22-11-04625.2002 PMC6758792

[69] Sillito A, Jones H (1996) Context-dependent interactions and visual processing in v1. Journal of Physiology-Paris 90: 205–209. 10.1016/s0928-4257(97)81424-6 9116668

[70] Fairhall AL, Lewen GD, Bialek W, de Ruyter van Steveninck RR (2001) Effciency and ambiguity in an adaptive neural code. Nature 412: 787–792. 11518957 10.1038/35090500

[71] Martinez-Conde S, Macknik SL, Troncoso XG, Hubel DH (2009) Microsaccades: a neurophysiological analysis. Trends in Neurosciences 32: 463–475. 19716186 10.1016/j.tins.2009.05.006

[72] Panzeri S, Petersen RS, Schultz SR, Lebedev M, Diamond ME (2001) The role of spike timing in the coding of stimulus location in rat somatosensory cortex. Neuron 29: 769–777. 11301035 10.1016/s0896-6273(01)00251-3

[73] Wolfe J, Houweling AR, Brecht M (2010) Sparse and powerful cortical spikes. Current Opinion in Neurobiology 20: 306–312. 20400290 10.1016/j.conb.2010.03.006

[74] Kumar A, Rotter S, Aertsen A (2010) Spiking activity propagation in neuronal networks: reconciling different perspectives on neural coding. Nat Rev Neurosci 11: 615–627. 20725095 10.1038/nrn2886

[75] van Vreeswijk C (2000) Analysis of the asynchronous state in networks of strongly coupled oscillators. Phys Rev Lett 84: 51105113. 10.1103/PhysRevLett.84.5110 10990879

[76] Ermentrout B, Pascal M, Gutkin B (2001) The effects of spike frequency adaptation and negative feedback on the synchronization of neural oscillators. Neural Computation 13: 1285–1310. 11387047 10.1162/08997660152002861

[77] Britten KH, Shadlen MN, Newsome WT, Movshon JA (1993) Responses of neurons in macaque MT to stochastic motion signals. Visual neuroscience 10: 1157–1169. 8257671 10.1017/s0952523800010269

[78] Malina KCK, Jubran M, Katz Y, Lampl I (2013) Imbalance between excitation and inhibition in the somatosensory cortex produces postadaptation facilitation. The Journal of Neuroscience 33: 8463–8471. 23658183 10.1523/JNEUROSCI.4845-12.2013 PMC6619615

[79] Wang HP, Spencer D, Fellous JM, Sejnowski TJ (2010) Synchrony of thalamocortical inputs maximizes cortical reliability. Science 328: 106–109. 20360111 10.1126/science.1183108 PMC2859205

[80] Shinomoto S, Kim H, Shimokawa T, Matsuno N, Funahashi S, et al (2009) Relating neuronal firing patterns to functional differentiation of cerebral cortex. PLoS Comput Biol 5: e1000433. 19593378 10.1371/journal.pcbi.1000433 PMC2701610

[81] Rolls ET, Treves A (2011) The neuronal encoding of information in the brain. Progress in Neurobiology 95: 448–490. 21907758 10.1016/j.pneurobio.2011.08.002

[82] Häusler C, Susemihl A, Nawrot MP (2013) Natural image sequences constrain dynamic receptive fields and imply a sparse code. Brain Research 10.1016/j.brainres.2013.07.056 23933349

[83] Shusterman R, Smear MC, Koulakov AA, Rinberg D (2011) Precise olfactory responses tile the sniff cycle. Nature Neuroscience 14: 1039–1044. 21765422 10.1038/nn.2877 PMC13348895

[84] Nawrot MP (2012) Dynamics of sensory processing in the dual olfactory pathway of the honeybee. Apidologie 43: 269–291.

[85] Turner GC, Bazhenov M, Laurent G (2008) Olfactory representations by drosophila mushroom body neurons. Journal of Neurophysiology 99: 734–746. 18094099 10.1152/jn.01283.2007

[86] Moreaux L, Laurent G (2008) A simple method to reconstruct firing rates from dendritic calcium signals. Frontiers in Neuroscience 2: 176–185. 19225590 10.3389/neuro.01.032.2008 PMC2622756

[87] Assisi C, Stopfer M, Laurent G, Bazhenov M (2007) Adaptive regulation of sparseness by feedforward inhibition. Nat Neurosci 10: 1176–84. 17660812 10.1038/nn1947 PMC4061731

[88] Papadopoulou M, Cassenaer S, Nowotny T, Laurent G (2011) Normalization for sparse encoding of odors by a wide-field interneuron. Science 332: 721–725. 21551062 10.1126/science.1201835 PMC3242050

[89] Gupta N, Stopfer M (2012) Functional analysis of a higher olfactory center, the lateral horn. Journal of Neuroscience 32: 8138–8148. 22699895 10.1523/JNEUROSCI.1066-12.2012 PMC3391592

[90] Jortner RA, Farivar SS, Laurent G (2007) A simple connectivity scheme for sparse coding in an olfactory system. The Journal of Neuroscience 27: 1659–1669. 17301174 10.1523/JNEUROSCI.4171-06.2007 PMC6673743

[91] Jortner RA (2013) Network architecture underlying maximal separation of neuronal representations. Frontiers in Neuroengineering 5: 19. 23316159 10.3389/fneng.2012.00019 PMC3539730

[92] Caron SJC, Ruta V, Abbott LF, Axel R (2013) Random convergence of olfactory inputs in the drosophila mushroom body. Nature 497: 113–117. 23615618 10.1038/nature12063 PMC4148081

[93] Strube-Bloss MF, Nawrot MP, Menzel R (2011) Mushroom body output neurons encode odorreward associations. J Neurosci 31: 3129–3140. 21414933 10.1523/JNEUROSCI.2583-10.2011 PMC6623757

[94] Lüdtke N, Nelson ME (2006) Short-term synaptic plasticity can enhance weak signal detectability in nonrenewal spike trains. Neural computation 18: 28792916. 10.1162/neco.2006.18.12.2879 17052149

[95] Ratnam R, Nelson M (2000) Nonrenewal statistics of electrosensory afferent spike trains: implications for the detection of weak sensory signals. J Neurosci 20 (17) 6672–6683. 10964972 10.1523/JNEUROSCI.20-17-06672.2000 PMC6772956

[96] Tsodyks M, Uziel A, Markram H (2000) Synchrony generation in recurrent networks with frequency-dependent synapses. J Neurosci 20: 50. 10.1523/JNEUROSCI.20-01-j0003.2000 PMC6774142 10627627

[97] Loebel A, Tsodyks M (2002) Computation by ensemble synchronization in recurrent networks with synaptic depression. J Comput Neurosci 13: 111–24. 12215725 10.1023/a:1020110223441

[98] Puccini GD, Sanchez-Vives MV, Compte A (2007) Integrated mechanisms of anticipation and rate-of-change computations in cortical circuits. PLoS Comput Biol 3: e82. 17500584 10.1371/journal.pcbi.0030082 PMC1866356

[99] Markram H, Wang Y, Tsodyks M (1998) Differential signaling via the same axon of neocortical pyramidal neurons. Proceedings of the National Academy of Sciences 95: 5323–5328. 10.1073/pnas.95.9.5323 PMC20259 9560274

[100] Thomson AM, Deuchars J, West DC (1993) Large, deep layer pyramid-pyramid single axon EPSPs in slices of rat motor cortex display paired pulse and frequency-dependent depression, mediated presynaptically and self-facilitation, mediated postsynaptically. Journal of Neurophysiology 70: 2354–2369. 8120587 10.1152/jn.1993.70.6.2354

[101] Treves A (1993) Mean-field analysis of neuronal spike dynamics. Network: Computation in Neural Systems 4: 259284.

[102] Shriki O, Hansel D, Sompolinsky H (2003) Rate models for conductance-based cortical neuronal networks. Neural computation 15: 18091841. 10.1162/08997660360675053 14511514

[103] Moreno-Bote R, Renart A, Parga N (2008) Theory of input spike auto- and cross-correlations and their effect on the response of spiking neurons. Neural Comput 20: 1651–705. 18254697 10.1162/neco.2008.03-07-497

[104] Moreno R, de la Rocha J, Renart A, Parga N (2002) Response of spiking neurons to correlated inputs. Physical Review Letters 89: 288101. 12513181 10.1103/PhysRevLett.89.288101

[105] van Vreeswijk C (2010) Stochastic models of spike trains. In: Analysis of Parallel Spike Trains, Springer, Springer Series in Computational Neuroscience. pp. 3–20.

[106] Gewaltig MO, Diesmann M (2007) NEST (NEural simulation tool). Scholarpedia 2: 1430.

[107] Chou YH, Spletter ML, Yaksi E, Leong JCS, Wilson RI, et al (2010) Diversity and wiring variability of olfactory local interneurons in the drosophila antennal lobe. Nature Neuroscience 13: 439–449. 20139975 10.1038/nn.2489 PMC2847188

